# The *Mycoplasma hyorhinis* genome displays differential chromatin accessibility

**DOI:** 10.1016/j.heliyon.2023.e17362

**Published:** 2023-06-15

**Authors:** Lewis Taylor, Steven Walsh, Anna Ashton, Norbert Varga, Sejal Kapoor, Charlotte George, Aarti Jagannath

**Affiliations:** a Sleep and Circadian Neuroscience Institute (SCNi), Nuffield Department of Clinical Neurosciences, University of Oxford, New Biochemistry Building, , South Parks Road, Oxford, OX1 3QU, UK; b MRC Weatherall Institute of Molecular Medicine, Radcliffe Department of Medicine, John Radcliffe Hospital, Oxford, OX3 9DU, UK

**Keywords:** Mycoplasma, Chromatin, Accessibility, Transcription

## Abstract

Whilst the regulation of chromatin accessibility and its effect on gene expression have been well studied in eukaryotic species, the role of chromatin dynamics and 3D organisation in genome reduced bacteria remains poorly understood [1,2]. In this study we profiled the accessibility of the *Mycoplasma hyorhinis* genome, these data were collected fortuitously as part of an experiment where ATAC-Seq was conducted on *mycoplasma,* contaminated mammalian cells. We found a differential and highly reproducible chromatin accessibility landscape, with regions of increased accessibility corresponding to genes important for the bacteria's life cycle and infectivity. Furthermore, accessibility in general correlated with transcriptionally active genes as profiled by RNA-Seq, but peaks of high accessibility were also seen in non-coding and intergenic regions, which could contribute to the topological organisation of the genome. However, changes in transcription induced by starvation or application of the RNA polymerase inhibitor rifampicin did not themselves change the accessibility profile, which confirms that the differential accessibility is inherently a property of the genome, and not a consequence of its function. These results together show that differential chromatin accessibility is a key feature of the regulation of gene expression in bacteria.

## 1 Introduction

In both prokaryotic and eukaryotic organisms, the organised compaction of the genome is necessary for its packaging into a small volume, whilst allowing functional accessibility for DNA-based processes. Three-dimensional organisation of the genome in eukaryotes as a result of nucleosomal patterning around histones is key to differential gene regulation. Increased accessibility is generally seen in nucleosome-free regions, such as seen around promoters and transcription start sites, whereas chromatin inaccessibility correlates with gene repression [3,4]. The equivalent 3D organisation that allows functional gene regulation in bacteria remains poorly understood [1,2]. In general, the bacterial genome has a hierarchical structure, comprising of a single circular chromosome which is highly condensed to form the nucleoid, although multiple exceptions have been reported [5]. Bacterial genomes are relatively gene dense, lack intronic spaces and have short intergenic regions, which contrasts with eukaryotic genomes, in which up to 99% of the genome is non-coding. Furthermore, in bacteria, such 3D organisation may be achieved through several factors, including DNA supercoiling, molecular crowding and nucleoid-associated proteins (NAPs), which act in poorly defined and fairly heterogenous ways to shape chromatin architecture [6]. The activities of NAPs are analogous to eukaryotic histone proteins, they bind to DNA to organise it into various conformations through DNA bridging, bending, looping or wrapping architecture [6,7]. A greater understanding of bacterial chromosome organisation has been achieved using various chromosome conformation capture approaches, which have shown clear topological and subcellular domains [2,8]. However, the net result of such organisation on bacterial chromatin accessibility and its bidirectional relationship with transcriptional patterns are not clearly understood.

Mycoplasma is most often encountered in the research setting as an unwanted contamination in mammalian culture, where it has been remarkably successful. Mycoplasma rarely results in overt changes in appearance or physiology, thus estimates suggest anywhere between 10 and 85% of cell lines commonly used in the laboratory carry a mycoplasma infection that is not evident until specifically assayed for. Indeed, our study was conducted under such conditions, and thus captures the biology of mycoplasma where it is thriving.

## 2 Experimental model and subject details

### 2.1 In vitro cell culture studies

Human U2OS cells and murine NIH3T3 cells were obtained from ATCC (ATCC® HTB-96™ and ATCC® CRL-1658™ respectively), maintained the lab for >5 years, and cultured in complete DMEM (DMEM supplemented with 10% FCS and 1% penicillin/streptomycin) at 37 °C, 5% CO_2_ as described in Taylor et al. [9]. Cells were lifted by incubation with TrypLE express for 5 min, diluted with complete DMEM and counted by trypan blue exclusion. Cells were either passaged into new tissue culture flasks, or plated into multi well plates for *in vitro* experiments. *Mycoplasma hyrorhinis* contamination was noticed upon ATAC-Seq on these cells.

### 2.2 ATAC-Seq library preparation

100,000 cells lifted with Tryple-E and were pelleted at 400 g for 5 min. Cells were lysed using a dounce homogenizer (Sigma, D8938) in 1 ml lysis buffer (20 mM Tricine-KOH, 25 mM MgCl2, 0.25 M sucrose, 1 mM DTT, 0.15 mM spermine, 0.5 mM spermidine, 0.3% IGEPAL-630, EDTA-free protease inhibitor) for 10 strokes. The homogenate was filtered through a 40 μm strainer into a 50 ml falcon tube, transferred to a 1.5 ml eppendorf tube, underlaid with a cushion buffer (0.5 mM MgCl2, 0.5 mM DTT, EDTA-free protease inhibitor, 0.88 M sucrose), and centrifuged at 3000 g for 20 min at 4 °C. The pellet was washed in 500 μl cold PBS and re-pelleted at 700 g for 10 min. The pellet was resuspended in transposition reaction mix (25 μL 2× TD Buffer (Illumina Cat #FC-121-1030); 2.5 μL Tn5 Transposase (Illumina Cat #FC-121-1030); 22.5 μL Nuclease Free H2O) and incubated for 30 min at 37 °C for transposition to occur. DNA was then purified using the Qiagen MinElute Kit and eluted with 23 μL warm 10 mM Tris pH8. The fragments were amplified, and Illumina adapters added by preparing the following mix (10 μL Transposed DNA; 10 μL Nuclease Free H_2_O; 2.5 μL 25 M Customized Nextera PCR Primer 1 Barcodes given in supplementary data); 2.5 μL 25 μM Customized Nextera PCR Primer 2 (Sigma Barcode Ad1_noMX); 25 μL Fast Sybr Green 2× PCR Master Mix (Qiagen Cat #4385612)) and subjected to 15–17 cycles of steps 3–5 ((1) 72 °C, 5 min; (2) 98 °C, 30 s (3) 98 °C, 10 s; (4) 63 °C, 30 s; (5) 72 °C, 1 min; Libraries quantified using the KAPA library quantification kit (KK4824), pooled and diluted to 20pM, and sequenced on the Illumina NextSeq 500 system with a 70 cycle high output cartridge.

### 2.3 DNA extraction and qPCR

Genomic DNA (plus *Mycoplasma* genomic DNA) was extracted from cells using the Qiagen DNeasy Blood and Tissue Mini Kit following the manufacturer's instructions. DNA concentration and quality was determined using a NanoDrop ND-1000 spectrophotometer qPCR was then conducted using 100 ng of template DNA using the Quantifast SYBR Green PCR Kit and a StepOnePlus thermal cycler (Applied biosystems) with the following thermal profile: 95 °C for 5 min and then 40 cycles of 95 °C for 10s, 60 °C for 30s and 72 °C for 12 s. Quantification of transcript levels was conducted using the relative standard curve method, for comparing within a gene, or the 2^−ΔΔCt^ method for comparison across genes. Primer sequences used can be found in Supplementary Table 1.

### 2.4 RNA sequencing library preparation

Following total RNA extraction using the Qiagen RNeasy mini kit, RNA sequencing libraries were prepared using the Illumina TruSeq Stranded Total RNA library prep gold kit following the manufacturer's instructions and 150 ng of starting material as described in Taylor et al. [9]. Briefly, 150 ng of total RNA was depleted of ribosomal and mitochondrial ribosomal RNA, cleaned up using RNA clean XP beads (Beckman Coulter, High Wycombe, United Kingdom) and then fragmented. Next, first and second stand cDNA synthesis was conducted, and the resultant cDNA purified using AMPure XP beads (Beckman Coulter) and adenylated at the 3′ end. Illumina indexing adapters were then ligated to the cDNA, the fragments purified and then enriched by PCR. Following amplification, the libraries were purified and then their concentration determined using the KAPA Library Quantification Kit for Illumina Platforms following the manufacturer's instructions. The libraries were then diluted to 4 nM and pooled in equal volumes prior to sequencing. Paired end RNA sequencing was then conducted using the NextSeq 550 and a Nextseq 500/500 v2 75 cycle kit, with the library loaded at 1.8 pM.

### 2.5 ATAC-Seq and RNA sequencing data analysis

The raw reads were initially processed to remove adapter sequences and trim low quality ends. A reference genome sequence and annotation combining human (GRCh38) with *Mycoplasma hyrorhinis* (NC_014448 from the PATRIC Bacterial and Viral Bioinformatics Resource Center) was prepared. The ATAC-Seq reads were mapped using HISAT2 and quantified with featurecounts. The RNA-Seq reads were mapped using Salmon and gene counts generated using Kallisto, normalisation and differential expression were conducted on DeSeq2 (version 1.16). Coverage was determined using BAMCoverage (deepTools2.0) with which normalised wiggle plots were generated. Standard deepTools normalisation was applied here using the BPM (number of reads per bin/sum of all reads per bin in millions) option.

## 3 Results and discussion

### 3.1 The mycoplasma genome displays a differential chromatin accessibility landscape

We performed Assay for Transpose Accessible Chromatin Sequencing (ATAC-Seq - [10]) on mouse NIH3T3 fibroblast cells as part of a separate study and discovered upon data analysis that a large number of reads did not map to the human genome (∼40%). A blast search showed matches to the *Mycoplasma hyorhinis* HUB-1 genome instead, revealing a mycoplasma infection. This presented an opportunity to investigate the genome accessibility profile in this bacterial species whilst it was contained within its host cell line. The *Mycoplasma* genus of bacteria have a parasitic lifestyle and several species are pathogenic to their animal and human hosts. They are self-replicating organisms with the smallest reduced genome (0.8 Mbp for *Mycoplasma hyorhinis* HUB-1), which is accompanied by a considerably reduced suite of transcription factors and NAPs [11,12].

**Fig. 1:**
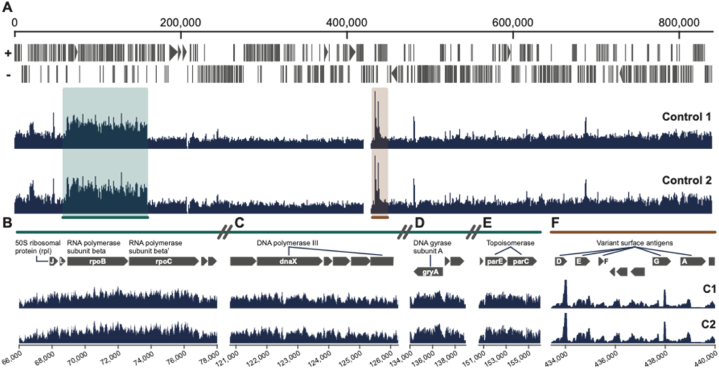
The *Mycoplasma* genome differential DNA accessibility landscape. (A) ATAC-Seq data visualisation across the *Mycoplasma hyorhinis* genome. Regions of interest containing genes essential for the bacterial life cycle highlighted in green and brown, including (B) *rplJ* and *L* – 50S ribosomal proteins, *rpoB* and *rpoC* – RNA polymerase subunits, (C) *dnaX* – DNA polymerase III, (D) *gryA* – DNA gyrase subunit A, (E) *parC* and *parE* – Topoisomerases, and (F) *vlpA*, *D*, *E*, *F* and *G* – Variant surface antigens. Data are from two independent biological replicates (Control 1 and 2) and are displayed as normalised (using bedtools) ATAC counts. The Y-axis on all wiggle plots is group scaled number of reads. (For interpretation of the references to colour in this figure legend, the reader is referred to the Web version of this article.)

Whilst we expected to see uniform accessibility across the genome to Tn5 as part of the ATAC-Seq approach, we were surprised to see regions of markedly increased accessibility (Fig. 1A. One region that showed two to three-fold increased reads over surrounding areas contained genes important to the viral life cycle (green region – Fig. 1A), and a selection of these including ribosomal subunits, DNA polymerase III and topoisomerases are illustrated (*rplJ, rplL, rpoB, rpoC* – Fig. 1B; *dnaX* – Fig. 1C; *gryA* – Fig. 1D; *parC, parE* – Fig. 1E respectively). Another region that showed eight to ten-fold increased reads over surrounding regions contained the surface antigens – *vlpA, D, E, F* and *G* (Fig. 1F), which are responsible for antigenic diversity for host adaptation). Our results are in agreement with some previous studies. It has been reported that the *Mycoplasma* chromosome has a defined 3D structure as in larger bacteria [13], with Hi-C (a high-throughput genomic technique to capture chromatin conformation) and super-resolution microscopy demonstrating three-dimensional chromosome organisation into 15–30 kb domains, with genes within the same domain being coregulated. Furthermore, the above study and others suggest local chromosome organisation is achieved through DNA supercoiling [14]. However, 5C analysis (similar to Hi-C) of the *Caulobacter crescentus* genome showed that repositioning elements of the chromosome such that the subcellular location of genes changed, did not alter gene expression [15]. Therefore the functional consequence of such 3D organisation remains unclear. Furthermore, *in silico* approaches suggest differential chromatin accessibility could indeed be a property of bacterial genomes that determines transcription factor occupancy [16], although other studies suggest transcription factor binding is determined by affinity for the sequence alone [17]. Our results confirm that accessibility across the *Mycoplasma* genome is indeed differential, but how this correlated with gene function and transcription were not clear.

### 3.2 The differential accessibility profile correlates with gene organisation

**Fig. 2:**
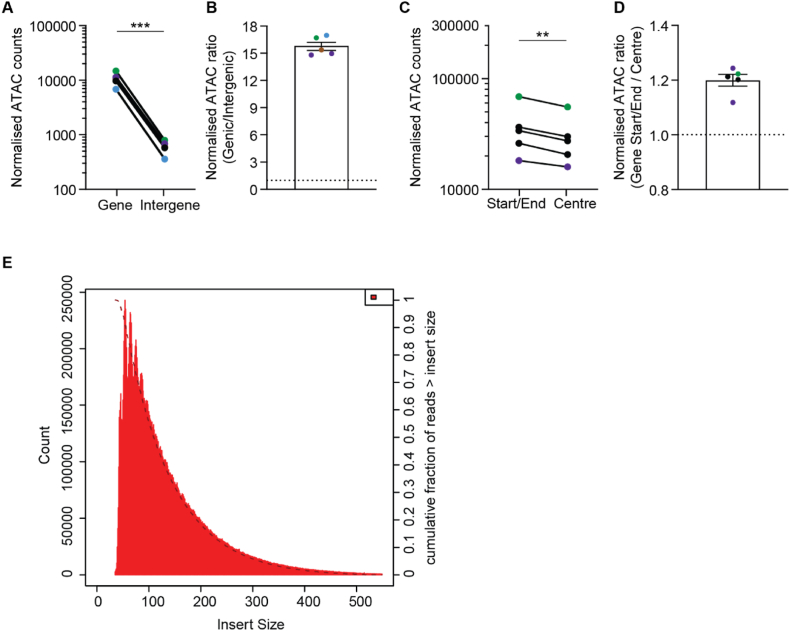
The differential DNA accessibility of the *Mycoplasma* genome correlates with gene organisation. (A) Quantification of normalised ATAC-Seq counts at gene versus intergene regions across five samples and (B) the fold change of genic versus intergenic counts. (C) Quantification of normalised ATAC counts at the start or end vs the centre of a gene and (D) the fold change of gene start/end vs gene centre counts (A–D) data are mean only n = 5 independent samples. For (A and C) statistical analysis was conducted by a two-tailed ratio paired *t*-test. ns P > 0.05, **P < 0.01 ***P < 0.001. (E) Fragment size distribution from one ATAC-Seq sample showing peaks distributed every 9–10 bases, with most fragments being 35–100 bp long. Error bars are standard error of mean.

To assess whether the patterns of accessibility had any correlation with the functional organisation of the genome, we first quantified the ATAC-Seq reads at within genes versus in the intergenic regions (Fig. 2A) and found that overall, there was a close to fifteen-fold difference in the number of counts (Fig. 2B), indicating far greater accessibility within the coding regions. This is in contrast to the findings of Gomes et al., where intergenic regions were more accessible than protein-coding [16]. However, the non-coding regions of the *Mycoplasma* genome are fairly sparse, as is the case for most prokaryotes, therefore our results may be influenced by the relatively low sample size for intergenic regions. Furthermore, increased accessibility where there is protein-DNA interaction is a key feature of genome organisation. We predicted that transcription factor binding at promoters [3], transcriptional machinery at start sites and the stalling/slowing down of transcriptional machinery at the end of genes [18] would cause increased accessibility at the start and end of genes. Therefore we assessed the number of ATAC-Seq counts at the start and end of each gene, defined as 10% of the gene at each end, and the centre of the gene, which covers the remaining 80%. Counts were normalised for the length of each gene. This showed a more modest, but also statistically significant, enrichment of reads at the start/end of a gene versus its centre (Fig. 2C and D). Finally, we quantified the percentage of A or T bases in the regions with 50 or greater ATAC-Seq reads (Top), versus those with less (Rest). The Top regions were 78.78% AT (standard deviation 9.027), whilst the Rest were 71.99% AT (standard deviation 8.08) indicating the regions showing greater accessibility may have fractionally greater AT content, although this is not statistically significant. Finally, we measured read fragment lengths and found a large spread from 35 to 500 bases (Fig. 2E), with peaks occurring 10 bases apart. Histone like proteins, the most conserved and abundant of which is HU [1] have been described in many bacterial species including *Mycoplasma* [19], with the *Escherichia coli* HU protein appearing to bind DNA as dimer at 9 base-pair intervals apart regardless of sequence [20]. Therefore, it is tempting to speculate that this is similar to nucleosomal patterning seen in ATAC-Seq data from eukaryotes, where fragments occur in peaks approximately 200 bases apart. Together, these results indicate the differential accessibility pattern may correlate with the functional organisation of the *Mycoplasma* genome.

We then assessed a number of possibilities that the data observed were artefacts of the model or experimental technique. A key feature of the genes that code for surface antigens is a variable number of repeats contained within the sequence. As such, it is possible that the increased reads we found in these regions corresponded to multiple repeats in the reads mapping to a single repeat in the reference genome. This was not apparent in the mapped data, where we saw unique reads across the different repeats (Supplementary Fig. 1). We next addressed whether copy number variation (whether multiple copies of the same gene were found in the genome) could underlie the difference in ATAC signal across the *Mycoplasma* genome. We designed PCR primers to cover discrete genomic regions (Supplementary Fig. 2A) in three genes of interest – *ushA*, *rpoB* and *vlpD.* A quantification of ATAC-Seq reads within these regions is indicated in Supplementary Fig. 2B. The corresponding signal from PCR product normalised to host (human) housekeeping genes is shown in Supplementary Fig. 2C, and these were not found to correspond to one another. Furthermore, the distribution of ATAC-Seq counts over the genes shows a large spread for some genes, but not others (Supplementary Fig. 2D).

### 3.3 Differential DNA accessibility correlates with levels of transcription, but is not modified by transcription

**Fig. 3:**
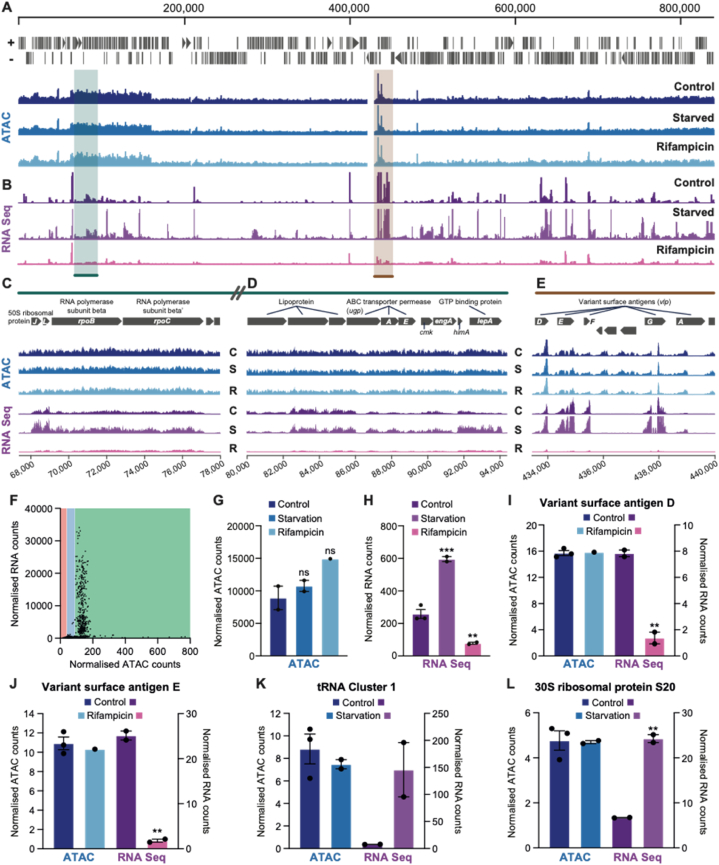
Differential DNA accessibility correlates with levels of transcription, but is not modified by transcription. *Mycoplasma* infected U2OS cells were either starved, treated with rifampicin (100 μM, 4 h), or kept under normal conditions (Control) and ATAC and RNA sequencing conducted. Visualisation of (A) normalised ATAC counts and (B) RNA sequencing counts across the genome. ATAC and RNA sequencing counts at genes essential for the *mycoplasma* lifecycle (C and D – Green region) and infectivity (E – Brown region). Y-axis is group-scaled number of reads. (F) Correlation analysis between normalised RNA and ATAC counts showing a threshold amount of DNA accessibility was needed to allow for gene expression (green area – permissive, red and blue – not permissive). Quantification of the average ATAC signal (F) and RNA sequencing counts (G) across the whole genome and at (I) variant surface antigen D and (J) E, (K) tRNA cluster 1 and (L) 30S ribosomal protein S20. For (F–L) data are mean ± SEM, n = 1–3 independent samples. (For interpretation of the references to colour in this figure legend, the reader is referred to the Web version of this article.)

We next sought to understand if the differential accessibility had any correlation with transcription. We performed RNA-Seq and ATAC-Seq on cells infected with *Mycoplasma* and mapped the *Mycoplasma* reads. We found overall that regions of higher accessibility did map to genes and also to regions of increased transcription (Fig. 3A and B). For example, the regions containing the RNA polymerase beta subunit genes (*rpoB* and *rpoC*) showed increased accessibility over surrounding regions, accompanied by increased transcription (Fig. 3C), as is also seen for a range of other genes including the variant surface antigens shown in Fig. 3D and E. However, there are clearly specific regions of where accessibility and levels of transcription do not correlate. This is illustrated (Supplementary Fig. 3) with the genes NADH oxidase and transposase IS861, where NADH oxidase appears more accessible, but it transcribed less. Furthermore, we observed high accessibility and transcription in a few regions where no genes were annotated (Supplementary Fig. 3). Interestingly, such findings have also been reported when profiling protein occupancy across the *E. coli* chromosome, where extensive occupancy was found at non-coding and transcriptionally silent areas, and these have been postulated to be organising centres that define topological domains in the bacterial genome [21].

Transcriptional activity shapes DNA organisation in many ways; active RNA synthesis induces nucleoid compaction and supercoiling, with negative supercoiling occurring upstream, and positive supercoiling occurring downstream of the transcription complex [22,23]. Additionally, the clustering of highly expressed genes into transcription foci defines chromosomal domain boundaries [24,25] The relationship between DNA organisation and gene expression is actually bidirectional, as chromosomal structure dynamics can regulate transcription through NAP-mediated changes in DNA topology and supercoiling, leading to changes in RNA polymerase and transcription factor binding [26]. Furthermore, 3D genome structure in bacteria is dynamic and influenced by growth conditions and stress [27,28]. Cagliero et al. used high-resolution chromosome conformation capture-based methods to analyse spatial organization of the *Escherichia coli* nucleoid and found dynamic 3D organisation, with replication causing greater interactions in the origin domains, and amino acid starvation causing spatial clustering of downregulated genes [28]. In order to assess whether changes in transcriptional activity were driving changes in accessibility in the *Mycoplasma* genome, we performed various treatments that would alter levels of transcription. These treatments involved starving the host cells by incubating in PBS or halting transcription by incubation with 100 μM rifampicin for 4 h each. Whilst these treatments clearly changed levels of transcription across the genome (Fig. 3B), no alterations in accessibility were seen (Fig. 3A).

An analysis of ATAC-Seq counts per base for variant surface antigen D/E (vlpD/E), the tRNA cluster and the 30S ribosomal protein S20 (*rpsT*) are illustrated in Fig. 3I/3J, 3K and 3L respectively, showing no change in accessibility in response to either starvation or rifampicin treatment, but a clear change in transcriptional responses, with starvation increasing transcription and rifampicin reducing it. Interestingly, some genes showed persistent expression even after rifampicin treatment (Fig. S3) and this may be due to longer degradation times for some transcripts. Several studies have tried to establish a possible role for transcription in chromosome organization using chemical inhibition of the initiation or elongation of RNA polymerase II (RNAPII) [29], and as detailed below. Some drugs used to inhibit transcription also affect RNAPII levels at the promoter, whereas others do not [29]. Therefore, when interpreting results from this type of experiment, it is important to consider whether only transcription has been affected or whether the presence of proteins in the transcription complex — which may be responsible for mediating interactions contact domains — has been affected too. In the bacteria *Caulobacter crescentus* (also known as *Caulobacter vibroides*) and *Bacillus subtilis*, inhibition of transcription using rifampicin results in a loss of contact domains [8,30]. Interestingly, conflicting results regarding the effect of rifampicin on nucleoid structure - both nucleoid expansion and nucleoid compaction - have been reported [22,31], likely due to differences in experimental conditions, analyses, and/or strains used. In our study, we saw no changes in accessibility profiles after treatment with rifampicin, but a clear drop in transcription.

We then we measured the correlation between the ATAC-Seq counts and corresponding RNA-Seq counts at the gene level (Fig. 3F), summarised across the genome in Fig. 3G and H. We noticed that there was little to no transcription where there was low accessibility (red and blue regions), confirming that accessibility was indeed required for a gene to be transcribed. However, once a threshold level of accessibility was reached (green region), levels of transcription did not correlate with levels of accessibility. This is in agreement with what is known about regulation of gene expression in eukaryotic systems, where accessibility at the promoter indicates “priming” or readiness for transcription, not necessarily levels of transcription. Together, these results indicate that the accessibility of chromatin in *Mycoplasma hyorhinis* is a requirement for gene expression, but is itself not modified by transcription.

A limitation of our study is that the conditions under which our experiments were performed were tailored to the culture of mammalian cells, and therefore may have been suboptimal for *Mycoplasma*. Indeed, the ATAC-Seq nuclei isolation and tagmentation protocol may not be optimal for capturing protein-DNA interactions in Mycoplasma. However, given the success of ATAC-Seq protocols in reliably capturing accessibility within mitochondrial DNA – indeed mitochondrial mapping reads are treated as an unavoidable experimental nuisance that have now been mined to provide useful accessibility data [32,33] - it is likely that the same applies to *Mycoplasma*. Furthermore, the extent of mycoplasma contamination across labs worldwide is a clear indicator that are conditions under which *Mycoplasma* thrive and are very successful, thus our observations are likely to underpin *Mycoplasma* gene regulation under more stringent conditions also.

In summary our data show that differential chromatin accessibility is a key feature of the regulation of gene expression in *Mycoplasma*. In general, it appears where there is no accessibility, there is no transcription, with genes that are key to the bacterial life cycle being the most highly accessible and transcriptionally active. Interestingly transcription itself does not alter accessibility, showing that it is not the dynamics of DNA coiling around RNA pol II that are responsible for the patterns seen. However, there are clear regions which are transcriptionally silent but highly accessible, suggesting this may be part of the mechanism by which complex topological organisation can be achieved [2,21].

## Author contribution statement

Lewis Taylor, Aarti Jagannath: Conceived and designed the experiments; Performed the experiments; Analysed and interpreted the data; Wrote the paper.

Steven Walsh: Conceived and designed the experiments; Performed the experiments; Analysed and interpreted the data.

Anna Ashton: Analysed and interpreted the data; Wrote the paper.

Norbert Varga, Sejal Kapoor: Performed the experiments.

Charlotte George: Analysed and interpreted the data.

## Funding statement

Dr Aarti Jagannath was supported by 10.13039/501100000268Biotechnology and Biological Sciences Research Council {BB/N01992X/1}.

## Data availability statement

Data are available at http://www.ncbi.nlm.nih.gov/bioproject/984476.

## Resource availability

### Lead contact

Further information and requests for resources and reagents should be directed to and will be fulfilled by Aarti Jagannath (aarti.jagannath@ndcn.ox.ac.uk)

### Materials availability

This study did not generate new unique reagents.

### Data and code availability

The datasets generated during this study will be deposited to the appropriate public repositories upon acceptance.

## Declaration of competing interest

10.13039/501100000769The authors declare that they have no known competing financial interests or personal relationships that could have appeared to influence the work reported in this paper.
