# Supplementary material for: The Mycoplasma hyorhinis genome displays differential chromatin accessibility
Source: Heliyon. 2023 Jun 15;9(6):e17362. doi: 10.1016/j.heliyon.2023.e17362 (PMC10300207; doi:10.1016/j.heliyon.2023.e17362)
Supplement: Multimedia component 1 [file mmc1.docx]

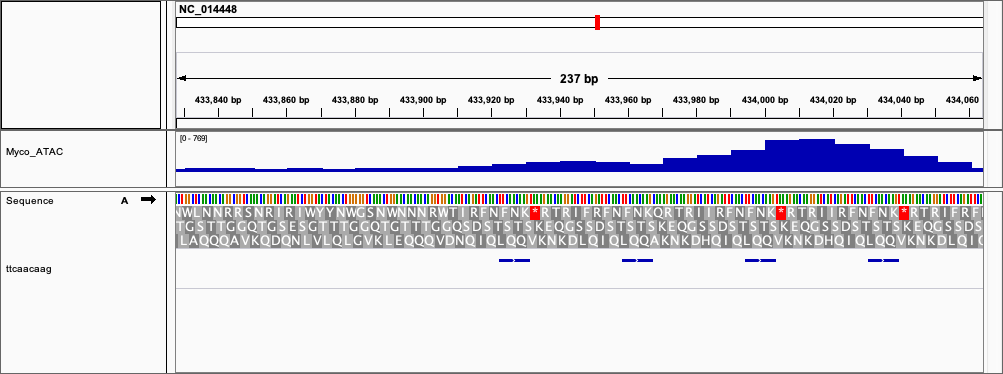


**Supplementary Figure 1.** ATAC-Seq data on the *vlpE* gene (Myco_ATAC) shown in blue, with the nucleotide sequence (t – red, a – green, g – yellow, c – blue) and three possible translation windows below – the middle window corresponds to the correct translation frame. The middle of 4 sequential 12-amino acid repeats indicated by the small blue bars below, showing unique reads mapping to the different repeats.


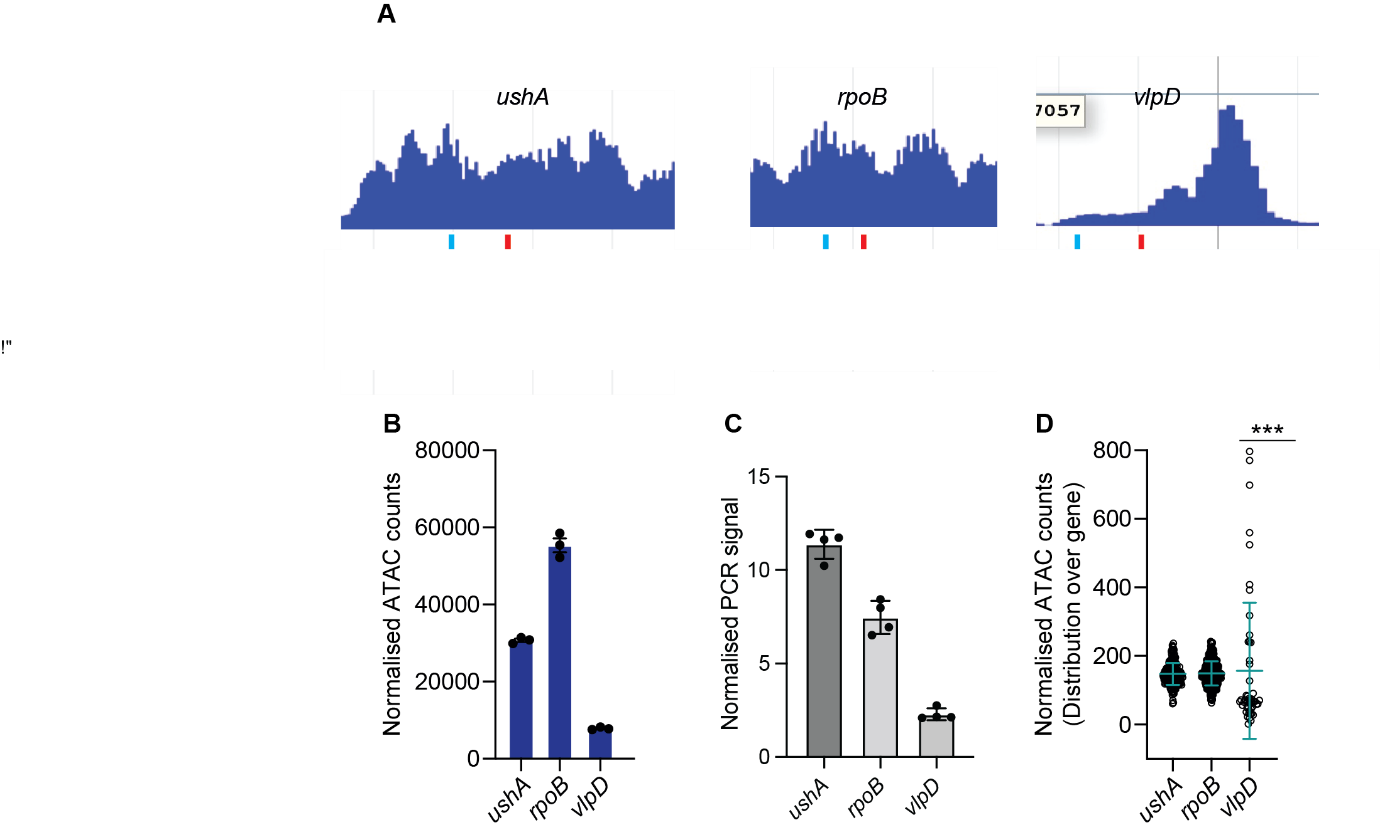


**Supplementary Figure 2.** **(A)** Positions of PCR primers on genes of interest overlaid on ATAC-Seq profile, blue indicates forward and red reverse. (**B**) Total ATAC-Seq counts for genomic regions analysed with qPCR in (**C)** where the amounts of gene specific products as indicated were normalised to three human housekeeping genes – GAPDH, ACTB and 18S, (**D**) The distribution of the density of ATAC-Seq reads over the same genes.


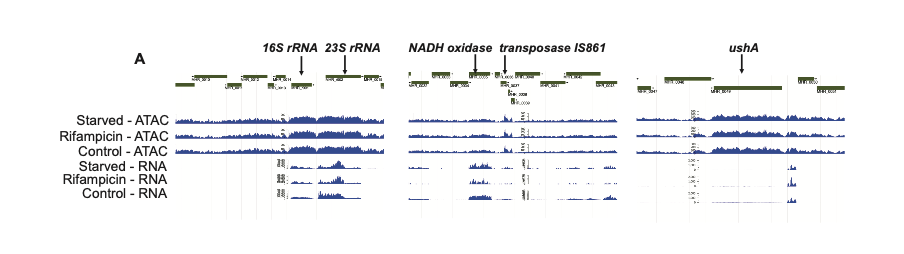


**Supplementary Figure 3.** **(A)** Data from ATAC-Seq (ATAC) and RNA-Seq (RNA) under the indicated conditions for three regions illustrating different relationships between accessibility and transcription. 16S and 23S rRNA are equally accessible, but 23S is transcribed more. Transposase IS861 is highly accessible, but not translated as is *ushA*, but the non-coding region next to ushA is highly transcribed.

**Supplementary Table 1. Primer sequences used to PCR genomic DNA**

vlpD F1: ACCACTGGAACTGGCTCAAC vlpD R1: TGTCCACCTGTTGTTGTTCC

UshA F1: AGCCATCACCACCTACTGAAAC UshA R1: TCCAGAGGAAGAAGTGGTGG

RpoB F1: TAACCGTGCGxsCTAATGGGTT RpoB R1: TCTCCATCTTCGCTAGCTCTG
